# Reply to “Acid inhibitors and allergy: comorbidity, causation and confusion”

**DOI:** 10.1038/s41467-020-17830-0

**Published:** 2020-08-07

**Authors:** Erika Jensen-Jarolim, Michael Kundi, Eva Untersmayr, Isabella Pali-Schöll, Berthold Reichardt, Galateja Jordakieva

**Affiliations:** 1 Institute of Pathophysiology and Allergy Research, Center of Pathophysiology, Infectiology and Immunology, Medical University of Vienna, Waehringer Guertel, 1090 Vienna, Vienna Austria; 2 The Interuniversity Messerli Research Institute, Medical University Vienna, University of Veterinary Medicine Vienna, University of Vienna, Veterinaerplatz 1, 1210 Vienna, Austria; 3 Center for Public Health, Medical University of Vienna, Kinderspitalgasse 15, 1090 Vienna, Austria; 4 Sickness Fund Burgenland, Siegfried-Marcus-Straße 5, 7000 Eisenstadt, Austria; 5 Department of Physical Medicine, Rehabilitation and Occupational Medicine, Medical University of Vienna, Waehringer Guertel, 1090 Vienna, Austria

**Keywords:** Allergy, Dyspepsia, Epidemiology, Risk factors

**Replying to** Kewin Tien Ho Siah *Nature Communications*10.1038/s41467-020-17831-z (2020).

We cordially thank Dr. Siah for their comments on our publication^1^, giving us the opportunity to clarify some points and to discuss limitations inherent in observational studies in general and analysis of claims data in particular.

Our publication “Country-wide medical records infer increased allergy risk of gastric acid inhibition”^2^ confirmed smaller previous studies in adults and children^3–5^. The relationship between anti-acid drug prescriptions followed by anti-allergy medication prescriptions was specific and not observed after prescriptions of other commonly prescribed drugs. With this in mind, we agree that the sequence of prescriptions is important and was among the first things we checked in our data base. However, it has to be considered that prescription rates of anti-acid drugs are around three times higher than anti-allergy medication prescriptions. It follows, that without any influence of an anti-allergy medication prescription on subsequent anti-acid drug prescriptions, this sequence must occur more frequently than the opposite one. However, our analyses of the country-wide and regional data sets showed that prescription of anti-allergy substances did not result in any elevated risk for subsequent anti-acid drug prescriptions. Contrarily, the hazard ratio for prescription of gastric acid-inhibiting drugs after anti-allergy drugs was below 1 (0.82 [95% confidence interval 0.80–0.84]; *p* < 0.001) when compared with other commonly prescribed drug classes used as controls, clearly implying an asymmetric relationship.

Considering the unidirectional phenomenon of an increased risk of anti-allergy medications after an anti-acid drug prescription, we agree that future research might reveal relationships linking dyspepsia and allergic diseases. Dr. Siah stressed correctly that observational studies and especially analyses of claims data, provide on their own no firm basis for cause-and-effect statements. Therefore, we referred the reader to the consistency of our findings with experimental data. Although we also agree that randomized controlled studies (RCTs) could, in principle, clarify whether or not the observed association is causal, we doubt that such RCTs will ever be conducted owing to a lack of commercial interest and because of the ethical issues.

As medical doctors, we regard gastric acid-inhibiting drugs as invaluable therapeutic options in a variety of dyspeptic diseases, if indication and reasonable prescription duration are considered. Also as MDs, we are trained to handle patients’ many anxieties, and must take the responsibility to explain, prescribe, and discontinue the anti-acid treatments in a controlled manner. We further stress that guidelines, pointing to a discontinuation of acid-inhibiting drug prescriptions as soon as there is no further indication, should be adhered to.

## Data Availability

The data that support the findings of this study were made available to the authors of this study by all major Austrian compulsory health insurance companies under the ethics vote ECS 1134/2014. General Data Protection Regulations Restrictions apply to the availability of these data, which were used under license for this study. However, any researcher can access the data by obtaining an ethical approval from the regional ethical review board and thereafter addressing requests for data to DI Berthold Reichardt, representative for the involved Austrian compulsory health insurance companies.

## Code availability

Most analyses were conducted using standard procedures without special code development. For data extraction and calculation of prevalences, code can be obtained upon request to the corresponding author.
